# Unintentional Injuries Among Young Adolescents at a Level-One Trauma Center in Saudi Arabia: A Cross-Sectional Study

**DOI:** 10.7759/cureus.36645

**Published:** 2023-03-24

**Authors:** Ibrahim Al Babtain, Yara Almalki, Nazish Masud, Deemah Asiri

**Affiliations:** 1 General Surgery, King Abdulaziz Medical City Riyadh, Riyadh, SAU; 2 General Surgery, King Abdulaziz Medical City Riyadh, Riyadh , SAU; 3 Biostatistics, Epidemiology and Environmental Health Sciences, Georgia Southern University, Statesboro, USA; 4 Medicine, King Saud Bin Abdulaziz University for Health Sciences, Riyadh, SAU

**Keywords:** saudi arabia, road traffic accidents, motor vehicle accidents, unintentional injury, adolescents, multiple trauma

## Abstract

Background

Unintentional injuries are the leading preventable cause of mortality across different demographics. This study aims to assess the prevalence, severity, contributing factors, and clinical outcomes of unintentional injuries among adolescent patients.

Methods

A retrospective study was conducted using the charts of patients admitted with unintentional injuries (motor vehicle accidents (MVA), falls, pedestrian injuries, burns, etc.) to the emergency department (ED) from January 2016 to December 2018 at a level-one trauma center in Riyadh, Saudi Arabia. A total of 721 patients’ charts were reviewed, but only 52 patients were consecutively included as per the definition of an adolescent. All variables, including severity and outcome, were assessed.

Results

The overall prevalence of unintentional injuries was 7.2 per 100 adolescent patients. The most common cause of unintentional injury were MVAs, which were reported in 35 (71%), with head and neck region injuries among 38 (73%) patients. The overall mortality was noted at 10 per 52 (19%) patients. The mean Injury Severity Score (ISS) score was 17.81±12.76. The patients who stayed longer in the ED were not associated with pelvic and lower extremity injuries, with a p-value=0.008. The ISS was the significant predictor of mortality, with an odds ratio (OR) of 1.6, a confidence interval (CI) of 1.02-2.65, and a p-value=0.04.

Conclusion

MVAs were the main cause of unintentional injuries among adolescents. Future recommendation plans for adolescents should include stricter implementation of road traffic laws to control this early, preventable death among adolescents.

## Introduction

The definition of an injury involves any resulting harm or damage to the body because of acute exposure to thermal, mechanical, electrical, or chemical energy. It can also occur due to a lack of heat or oxygen. Injury can either be intentional or unintentional; additionally, both are considered major causes of morbidity among adolescents in the United States [1,2]. Unintentional injuries may be further categorized by causes like motor vehicle accidents (MVAs), falls, burns, drowning, poisoning, choking, suffocation, and animal bites [1].

Among children and adolescents aged one to 19 years, unintentional injuries make up about 8.6 million visits to the emergency department (ED) and over 220,000 hospitalizations per year in the United States, and in 2017, adolescents contributed to 3.2 million ED visits [3]. Worldwide, about 830,000 deaths occur in children aged less than one to 14 years annually, and 2300 adolescents and children die daily [4,5]. Unintentional injuries are the leading cause of death in adolescents, making up 45.9% of all deaths that occur between the ages of 10 and 19 [1,3].

In 2004, the leading cause of death in Palestinian children was unintentional injuries, reported at 24.2% for ages one to four years and 29.2% for ages four to 19 years [6]. Another study done in Dubai was performed on students from grades seven to 12 and showed that unintentional school injuries (mainly falls and cuts) were more common, around 57.3%, among ages 12-14 years and that the incidence rate of these injuries was 297.7 per thousand [7].

A study done in Saudi Arabia showed that within the last 12 months, the prevalence of unintentional injuries in children was 24.7%, which was lower than other studies that reported a range of 35.8%-42.9% unintentional injuries among adolescents within the same time period, especially among the 13-15 age group [8-12]. Moreover, this study, being consistent with others, states that boys have a higher risk of injury and mortality [8-11,13,14]. Two studies in Saudi Arabia have reported a higher prevalence of unintentional injuries due to falls (62.9% and 31.9%, respectively), which is consistent with some studies that cite falls as being the most common cause of unintentional injury [7,13-15]. Others, however, count open cuts and wounds as the most common [16,17].

Currently, there are no recent studies that have been conducted in a level-one trauma center to further assess the prevalence of unintentional injury and the contributing factors among adolescents in Riyadh, Saudi Arabia.

## Materials and methods

Study design and settings

A retrospective study was conducted using the charts of patients admitted with multiple traumas to assess the prevalence of unintentional injury among trauma-activated adolescents. This study was conducted in the ED of King Abdulaziz Medical City (KAMC) in Riyadh, Saudi Arabia, a tertiary governmental level-one trauma center.

Participants

To identify the potential participants, charts of patients with multiple traumas were reviewed. Unintentional injuries were the focus of the study, which included MVAs, falls, pedestrian accidents, burns, etc., while intentional injuries that involved suicide or homicide were not part of this study. Patients were considered included for this study if they were adolescents ranging from 14-17 years old, presented with trauma activation, Saudi or non-Saudi, male or female, and those who died before undergoing tertiary survey or transferred from another hospital within the first 24 hours of the trauma. Patients were excluded if they died before adult ED arrival. The approximate population of unintentional injury patients in the adult ED at KAMC after trauma team activation was 2200 patients from January 2016 to December 2018 and was obtained from the healthcare center database. The sample size was 721 participants, and only 52 patients were adolescents as per the definition of adolescents and were sampled using consecutive sampling to include all possible unintentional injuries among adolescent patients brought to the ED [18].

Data sources 

Data were collected using a data collection sheet that extracted demographic and injury-related data from the electronic charts of all cases. Electronic chart data by two co-authors were reviewed by the principal investigator for accuracy. The two co-authors who collected all the data were not involved in treating the patients. A demographic sheet was used to evaluate the sociodemographic factors, including age, gender, and nationality. An injury-related sheet was used to extract the intubation status and its causes in all cases, the number of injuries, and the mechanisms of injury. The Glasgow Coma Scale (GCS), Revised Trauma Score (RTS), and Injury Severity Score (ISS) were calculated for all patients. The GCS is used to assess the motor, verbal, and eye responses, and the total score has values ranging from three being the lowest and showing a poorer prognosis to 15 being the highest to categorize the level of consciousness [19]. RTS is a scoring system used initially to estimate the severity and consists of the GCS, systolic blood pressure, and respiratory rate [20]. ISS uses the worst injury score in three separate body regions, in which the lower the sum score, the more severe the injury [21]. All radiological studies performed in the first 24 hours of hospital admission and after 24 hours of hospital admission, emergent operations within the first 24 hours, treatment after 24 hours of hospital admission, and hospital mortality were recorded. Circumstantial variables, which included the time of arrival to the ED and the length of hospital stay, were also collected.

Statistical analysis

Statistical analysis was performed using IBM's Statistical Package for the Social Sciences, version 22 (SPSS Inc., Chicago, IL). Descriptive statistics were employed in reporting frequencies and percentages for categorical variables such as the mechanism of injury, intubation status, and anatomical location of injuries, while mean and standard deviation were used for continuous variables such as age, GCS, RTS, and ISS. Inferential statistics using chi-square were used to assess the association of an emergency room (ER) length of stay of three or more hours with the prevalence of unintentional injuries in adolescent patients. A chi-square was also used to assess the association of injury-related characteristics with the prevalence of unintentional injuries. To assess the predictors of mortality with other variables, forward logistic regression was applied to show the correlation between the ISS and mortality rate. The p-value was set at 0.05 for all the tests applied.

Ethical approval 

Ethical approval of this study was obtained from the IRB RC20/062/R on September 16, 2021, and no medical record numbers were obtained to ensure confidentiality.

## Results

Demographic Characteristics

**Figure 1:**
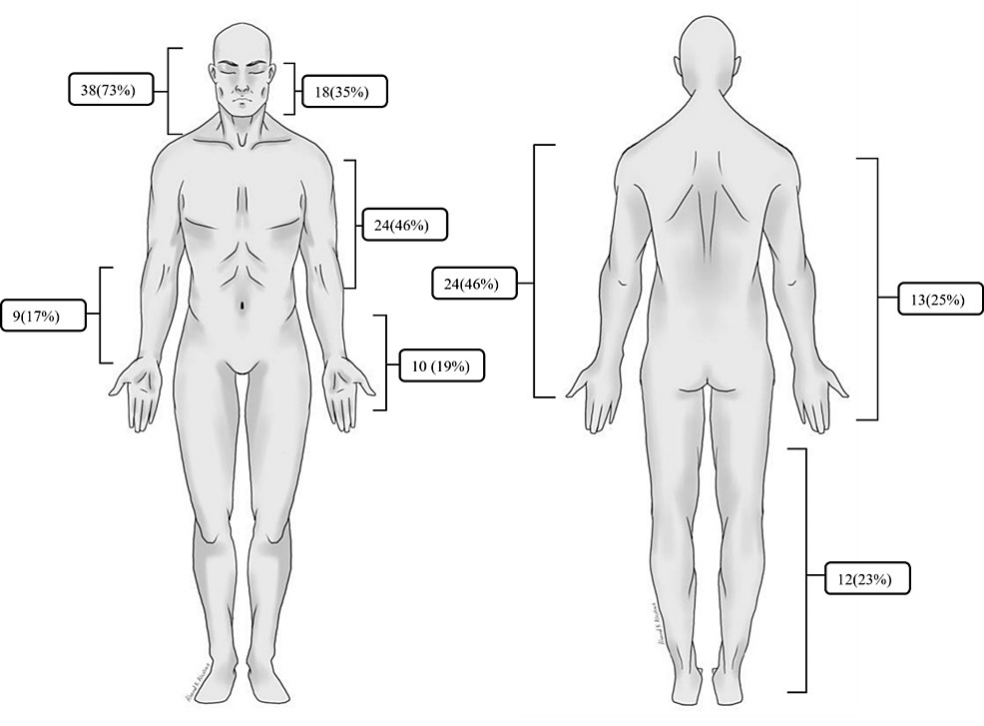
An illustration of the human body showing anatomical regions of unintentional injuries in adolescent patients This image has been created by Dr. Alanoud Alsubaie

The overall prevalence of unintentional injuries was 7.18 per 100 adolescent patients. A total of 52 patients were recruited for the study, with a mean age of 16.2±1.06, 47 (90%) of them being males. Overall mortality was noted in 10 (19%) patients. MVAs were reported as the most common mechanism of injury among 35 (67%) adolescent patients. Among 52 adolescent patients, 40 (77%) were intubated, with the highest cause of intubation being the loss of consciousness or traumatic brain injury (TBI) in 29 (83%) patients, with a mean number of days of intubation of 3.88±4.32 days. Thirty-eight (73%) patients reported having head and neck injuries, while 24 (46%) reported having spine injuries, and 24 (46%) had injuries in the thoracic cavity. GCS was reported among the 52 patients with a mean score of 9.13±4.46, RTS was reported with a mean score of 9.90±2.17, and ISS was reported with a mean score of 17.18±12.76 among adolescent patients. The length of stay in the ER in hours was reported with a mean of 4.59±5.52 hours. Among 52 adolescent patients, 20 (54%) were immediately transferred to the intensive care unit (ICU), followed by urgent surgery among 10 (19%) patients. On average, every patient had at least two injuries to different anatomical sites of the body, with a mean number of 2.15±3.37 as shown in Table 1 and Figure 1.

**Table 1:** Profile of adolescent patients with unintentional injuries (n=52) ER: emergency room; GCS: Glasgow Coma Scale; ICU: intensive care unit; ISS: injury severity score; MCA: motorcycle accident; MVA: motor vehicle accident; OR: operating room; RTS: revised trauma score; TBI: traumatic brain injury

Variables	Categories	Count	%
Age	Mean±SD	16.2± 1.06	
Nationality	Saudi	42	87%
	Non-Saudi	7	13%
Gender	Male	45	90%
	Female	4	10%
Mechanism of injury	MVA	35	67%
	MCA	3	6%
	Pedestrian	7	13%
	Falls	3	5%
	Other accidents	4	8%
Intubation status	No	12	23%
	Yes	40	77%
Causes of intubation	TBI	29	83%
	Chest injury	2	6%
	OR intubation	1	3%
	Other	3	9%
Number of injuries	1	36	21%
	2	9	27%
	3	6	12%
	4	4	8%
	5	7	13%
	6	7	13%
	7	1	2%
	8	1	2%
	10	1	2%
GCS	Mean±SD	9.10±4.46	
RTS	Mean±SD	9.87±2.17	
ISS	Mean±SD	18.20±12.76	
Hospital length of stay (days)	Mean±SD	22.85±43.44	
ER length of stay (hours)	Mean±SD	4.68±5.52	
ICU length of stay (days)	Mean±SD	9.27±9.08	
Intubation length of stay (days)	Mean±SD	4.11±4.32	
Number of injuries	Mean±SD	3.48±3.37	

Association of ER Length of Stay of Three Hours in Unintentional Injuries Among Adolescent Patients

Among the injury-related variables, intubation status, pelvic injuries, and lower extremity injuries were statistically significantly associated with an ER length of stay of more than three hours. With a p-value <0.04, this study showed that the 10 (83%) patients who had an ER length of stay of more than three hours were more likely to not be intubated. Furthermore, the 26 (65%) patients in this study who stayed in the ER for more than three hours were unlikely to have pelvic injuries with a p-value <0.04. For lower extremity injuries, a patient who had an ER length of stay of more than three hours had no lower extremity injuries; our study reported 26 (68%) patients with a p-value <0.008 (Table 2).

**Table 2:** Association of ER length of stay of three hours in unintentional injuries among adolescent patients (n=52) * The chi-square/ Fisher exact test as applicable significant at <0.05; ER: emergency room

		ER length of stay < 3 hours		ER length of stay ≥ 3 hours			
		Count	%	Count		%	P-value
Intubation status	Not intubated	2	16.70%	10	83.30%		P=0.04
	Intubated	19	50.00%	19	50.00%		
Pelvis	No	14	35.00%	26	65.00%		P=0.04
	Yes	7	70.00%	3	30.00%		
Lower extremities	No	12	31.60%	26	68.40%		P=0.008
	Yes	9	75.00%	3	25.00%		

Association of ISS and Mortality Rate

According to the logistic regression test, a high ISS was significantly associated with a higher mortality rate (r=0.53, odds ratio (OR)=1.6, confidence interval (CI): 1.02-2.65, and a p-value <0.04). 

## Discussion

Unintentional injuries are the leading cause of mortality across different populations. These injuries are widely preventable and have multiple implications for patients and healthcare workers [1-3]. This study aimed to assess the prevalence, severity, contributing factors, and clinical outcomes of unintentional injuries among adolescent patients.

In this study, the prevalence of unintentional injury was 7.18% among 52 adolescent patients who were admitted to the adult ED after trauma code activation in KAMC, marking a low-rate prevalence. In 2018, a similar rate was reported in a study conducted in India assessing the risk behaviors of unintentional injuries among school-aged adolescent boys, and among the participants, only 11.9% reported sustaining a serious unintentional injury in the past 12 months [22]. In comparison, higher prevalence rates were reported in multiple other studies [23,24]. Differences in population settings and external environmental factors could explain the observed differences in the prevalence rates.

In this current study, MVAs were the most common mechanism of injury among adolescents. In Jeeluna, a study on adolescent health in Saudi Arabia, 35.4% of adolescents reported having been involved in an MVA [24]. In our study, it was reported that 37 (or 65%) adolescents were drivers, even though the general Saudi road traffic law prohibits illegal drivers who are less than 18 years of age, which reflects the need for further law enforcement and monitoring. Head and neck injuries were the most common location of injuries among 38 (73%) adolescent patients in our study. Another extensive study that was conducted in the United States on primary and secondary school students reported that in school- and non-school-injured adolescents, the percentage of head and neck injuries was 26.4%-27.4%, respectively [25]. 

Non-intubated patients and patients with no pelvic or lower extremity injuries were less likely to stay in the ED for more than three hours in our study. On the other hand, intubated patients with critical injuries need urgent interventions, and it is unlikely that those patients will stay in the ED for more than three hours as they require immediate shifts to the ICU or OR [26]. The ISS was the sole predictor of mortality based on logistic regression among our population. The higher ISS was related to a 1.6 percent higher chance of mortality. Similar findings were reported in other studies [27,28].

On further assessment, the mortality rate was 19% among our population. It can be further explained that this high mortality rate is due to MVAs being the second leading cause of death in Saudi Arabia, reporting 32.2% of all adult mortality [29]. A recent study published about child mortality in Saudi Arabia reported a preventable cause of death for 172 (15%) patients, with MVAs accounting for 37 (80%) deaths among adolescents [30]. Thus, further implementation of stricter road traffic rules as a preventive measure is highly necessary.

Limitations

Some limitations were present in the study, one of which was that it was a cross-sectional study. National studies are often classified into age groups <15 or ≥15 years, thus adolescent data is mixed with adult data, making it difficult to get a full picture of adolescent data exclusively. In national practice, any patient >14 years that presents to the ED as an ‘adult’ and is managed by an adult ED physician in this study was considered a limitation. Another noted limitation was the study being conducted in one center due to the inability to acquire a comprehensive picture of the unintentional injuries in the adolescent population in Riyadh, Saudi Arabia.

Future studies can include data relating to the mechanism of injury in addition to the use of protective measures to obtain a clearer understanding of their role in the prevalence and type of injury in the Saudi pediatric and adolescent population.

## Conclusions

The overall prevalence of unintentional injuries in adolescents was low. The total percentage of deaths is high among adolescent patients, and almost all of them are due to MVAs. Furthermore, MVAs are one of the major causes of death among the young population. High ISS was the only predictor of mortality among adolescent patients. Stricter implementation of road traffic laws is highly needed to control this early loss among adolescents.
